# The First English Translations

**Published:** 1894-04-21

**Authors:** 


### The First Century of English Medical Literature.

II.—THE FIRST ENGLISH TRANSLATIONS.

It is rather remarkable that almost the first medical treatise which was translated into English was so done by an important dignitary of the Church, Thomas Paynell, Canon of Marten Abbey. The work which he selected was the "Regimen Sanitatis Salerni," which book he claims "is as profitable and as needful to be had and read as any can be to observe corporal health." Long before its appearance in English, in the year 1528, the "Regimen Sanitatis Salerni" had enjoyed a wide popularity. It appears to have been originally compiled for, and dedicated to, Robert, Duke of Normandy, son of William the Conqueror, who stayed at Salernum (where was an old-established medical university of high repute) for the cure of a wound received in Palestine. It is largely made up of extracts from Avicen and Galen, and has for its object the avoidance of drug taking by the use of a healthy and natural manner of life. The main directions are simplicity itself. To avoid great charges of thought and care (it was no doubt the fashion even then to talk of the high pressure of modern life); to avoid anger; to eat and drink soberly; to make a light supper, and eschew sleep after meat; such are the more general rules of life. Entering more into detail, we are urged to wash the eyes with clear cold water on rising; to perform the same office for our hands, and to observe that the washing of the hands in hot water after meals may lead to terrible consequences; to come a little hither and thither when we are risen from rest; to comb our head in the morning; to wash and purge the teeth; to stand upright after meals that the meat may descend to the bottom of the stomach, and then walk a little softly. To these rules are added many hints about diet, and the best seasons for letting blood. So mild a "regiment of health" attained immediate fame on its introduction to the unlearned in its English dress. It went through six editions before the end of the century, and then acquired new credit in a rhymed translation by Sir John Harrington, of which five editions were issued in a few years.

But Canon Paynell's services to medicine did not stop here. He prepared a translation of a small treatise on the use of the wood called Guiacum, recently found in "an ylonde named Spagnolo," written by that "great Almayne Ulrich Hutten, knyght," and furthermore issued "a much profitable treatise against pestilence." We shall have occasion later on to comment on the multiplicity of remedies confidently recommended as specifics for the plague. It will be sufficient to note from this rare and very early treatise the "pronosticate signes of pestilence," which may remind us of the persistent efforts made to trace the prevalence of influenza to the weather. The signs are "when that in the self-same summer's day the wind doth oftentimes vary and change; when that in summer the days do oftentimes appear obscure and dark, and as though it would rain, and yet it raineth not at all; when that in summer we see upon the earth great abundance of flies; when that the stars do appear and seem to fall and depart from their places; when that it seemeth to man that the comets or blazing stars do flee abroad; when there is much lightning proceeding out of the south part; when there bloweth much wind from the south part; the which is very noyfull and grievous to the people insomuch that none other medicine can help and succour them, but only the mercy of Almighty God." The superstitious dread of the south wind, arising in regions where the sirocco affects the health and spirits of every one, was indiscriminately adopted in northern countries, and is a marked feature in all the sanitary directions of this era.

Prognostications were long regarded as a regular part of the study of medicine. The question as to whether the patient shall live or die, to which the wisest hesitate in modern days to give answer, was then confidently solved by means founded half on observation, half on magic. The earliest example of this class of treatise is the Prognosticacion, printed in 1530, drawn out of the Books of Ipocras, Avicen, and other notable authors of physick, shewing the danger of divers sicknesses, that is to say whether peril of death be in them or not, the pleasure of Almighty God being reserved. The author confines himself to an enumeration of the graver symptoms occuring in various diseases, and likely to have a fatal termination. But "The Boke of Knowledge whether a sick person being in peril shall live or die," London, 1535, has a bolder scope. We are reminded of Falstaff's death* by the observation, "If the patient have thin nostrils, or the tip of his nose sharp . . . it signifieth soon death." A curious device was to "rub well the forehead of the sick with a tablet or other such of copper from the one ear to the other, and if he sleep after he shall live, or else not." A very deadly sign was "if the patient bid the physician farewell, unless in doing so he chance to put his hands to his head and draw up his feet, when he shall scape." A species of divination to know whether a man shall die within seven months or not was performed by letting a drop of his blood fall into a dish of water "If it descend whole to the bottom he shall live that year, and if not, but go abroad, not."

In the same year, 1535, was printed a book of impudent quackery, under the title of the "Prognostication for ever of Erra Pater, a Jew born in Jewry, a doctor in astronomy and physic, profitable to keep the body in health." He gives rules for every month in the year, consisting solely of the enumeration of certain days on which it is deadly to let blood, and such injunctions as "during this month, as oft as thou wilt drink good white wine fasting." There is a curious table at the end showing what will befall if it thunder on any given day of the week.

Two small compilations from Plutarch are among these early translations for the use of the unlearned. The one printed in 1530 is named "The Governaunce of Good Health," and aims at superseding the necessity for physic by a wise rule of life. The other, known as "Practica Plutarche," is full of curious prescriptions, of which one must suffice, "a powder against too much feebleness." It is brevity itself. "Take camphor, musk, of each three half-penny weight; shavings of ivory, of gold, of silver, of each three penny weight."

Some mention of a scarce and little known translation of (1530) Roger Bacon must conclude this notice of the first contributions to English medical literature. The book treats of the "best waters artificial, and the virtues and properties of the same, much profitable for the poor sick." The waters are of the most harmless description, many are distilled from flowers such as rosemary, red roses, lilies, &c. There is one which, with the wealthy, one can imagine exceedingly popular; it is made by taking wedges of fine gold well warmed in the fire and quenching them forty-two times within the water from a good well, which is to be drunk with wine or good ale. The admonition of the good friar at the end may have eased the consciences of some. "For to please and make the woman appear more fairer and younger unto their husbands it is suffered to use of some waters which maketh the visage fair and white."

* "Henry V.," Act ii., Scene 3.

